# Supplementary material for: Resolving the full spectrum of human genome variation using Linked-Reads
Source: Genome Res. 2019 Apr;29(4):635–45. doi: 10.1101/gr.234443.118 (PMC6442396; doi:10.1101/gr.234443.118)
Supplement: Supplemental Material [file supp_gr.234443.118_Supplemental_longranger-2.2.zip › longranger-2.2.tar.bz2!/lr2.2-public/lib/stan/cmdstan/stan/doc/api/html/functions_t.html]

Stan: Class Members


|  |  |
| --- | --- |
|  | Stan  2.5.0  probability, sampling & optimization |


- Main Page
- Related Pages
- Namespaces
- Classes
- Files

- Class List
- Class Index
- Class Hierarchy
- Class Members

- All
- Functions
- Variables
- Typedefs
- Enumerator
- Related Functions

- \_
- a
- b
- c
- d
- e
- f
- g
- h
- i
- j
- k
- l
- m
- n
- o
- p
- q
- r
- s
- t
- u
- v
- w
- x
- y
- z
- ~


All Classes Namespaces Files Functions Variables Typedefs Enumerations Enumerator Friends Macros Pages

Here is a list of all class members with links to the classes they belong to:

### - t -

- T()
  : stan::mcmc::base\_hamiltonian< M, P, BaseRNG >
  , stan::mcmc::dense\_e\_metric< M, BaseRNG >
  , stan::mcmc::diag\_e\_metric< M, BaseRNG >
  , stan::mcmc::unit\_e\_metric< M, BaseRNG >
- t0\_
  : stan::gm::integrate\_ode
  , stan::mcmc::stepsize\_adaptation
- T\_
  : stan::mcmc::base\_static\_hmc< M, P, H, I, BaseRNG >
- tangent()
  : stan::agrad::fvar< T >
- tau()
  : stan::mcmc::base\_hamiltonian< M, P, BaseRNG >
  , stan::mcmc::dense\_e\_metric< M, BaseRNG >
  , stan::mcmc::diag\_e\_metric< M, BaseRNG >
  , stan::mcmc::unit\_e\_metric< M, BaseRNG >
- term\_g
  : stan::gm::expression07\_grammar< Iterator >
- term\_grammar()
  : stan::gm::term\_grammar< Iterator >
- term\_r
  : stan::gm::term\_grammar< Iterator >
- theta\_
  : stan::gm::integrate\_ode
  , stan::math::coupled\_ode\_system< F, double, stan::agrad::var >
  , stan::math::coupled\_ode\_system< F, stan::agrad::var, stan::agrad::var >
- theta\_dbl\_
  : stan::math::coupled\_ode\_system< F, double, double >
  , stan::math::coupled\_ode\_system< F, double, stan::agrad::var >
  , stan::math::coupled\_ode\_system< F, stan::agrad::var, double >
  , stan::math::coupled\_ode\_system< F, stan::agrad::var, stan::agrad::var >
- thin
  : stan::io::stan\_csv\_metadata
- timing
  : stan::io::stan\_csv
- tinyness\_before
  : std::numeric\_limits< stan::agrad::fvar< T > >
  , std::numeric\_limits< stan::agrad::var >
- to\_column\_major()
  : stan::json::json\_data\_handler
- to\_var()
  : stan::agrad::OperandsAndPartials< T1, T2, T3, T4, T5, T6, T\_return\_type >
- to\_vec()
  : stan::io::var\_context
- tolAbsF
  : stan::optimization::ConvergenceOptions< Scalar >
- tolAbsGrad
  : stan::optimization::ConvergenceOptions< Scalar >
- tolAbsX
  : stan::optimization::ConvergenceOptions< Scalar >
- tolRelF
  : stan::optimization::ConvergenceOptions< Scalar >
- tolRelGrad
  : stan::optimization::ConvergenceOptions< Scalar >
- transition()
  : stan::mcmc::adapt\_dense\_e\_nuts< M, BaseRNG >
  , stan::mcmc::adapt\_dense\_e\_static\_hmc< M, BaseRNG >
  , stan::mcmc::adapt\_diag\_e\_nuts< M, BaseRNG >
  , stan::mcmc::adapt\_diag\_e\_static\_hmc< M, BaseRNG >
  , stan::mcmc::adapt\_unit\_e\_nuts< M, BaseRNG >
  , stan::mcmc::adapt\_unit\_e\_static\_hmc< M, BaseRNG >
  , stan::mcmc::base\_mcmc
  , stan::mcmc::base\_nuts< M, P, H, I, BaseRNG >
  , stan::mcmc::base\_static\_hmc< M, P, H, I, BaseRNG >
  , stan::mcmc::fixed\_param\_sampler
- traps
  : std::numeric\_limits< stan::agrad::fvar< T > >
  , std::numeric\_limits< stan::agrad::var >
- truncation\_
  : stan::gm::sample
- truncation\_range\_r
  : stan::gm::statement\_grammar< Iterator >
- ts\_
  : stan::gm::integrate\_ode
- type
  : add\_var::result< T1, T2, T3, T4, T5, T6 >
  , copy\_square\_cholesky\_dimension\_if\_necessary::result< T1 >
  , empty\_range::result< T1 >
  , set\_double\_range\_lower::result< T1, T2, T3, T4 >
  , set\_double\_range\_upper::result< T1, T2, T3, T4 >
  , set\_int\_range\_lower::result< T1, T2, T3, T4 >
  , set\_int\_range\_upper::result< T1, T2, T3, T4 >
  , stan::gm::add\_conditional\_body::result< T1, T2 >
  , stan::gm::add\_conditional\_condition::result< T1, T2, T3, T4 >
  , stan::gm::add\_expression\_dimss::result< T1, T2, T3, T4 >
  , stan::gm::add\_fun\_var::result< T1, T2, T3, T4 >
  , stan::gm::add\_function\_signature::result< T1, T2, T3, T4, T5 >
  , stan::gm::add\_loop\_identifier::result< T1, T2, T3, T4 >
  , stan::gm::add\_while\_body::result< T1, T2 >
  , stan::gm::add\_while\_condition::result< T1, T2, T3 >
  , stan::gm::addition\_expr3::result< T1, T2, T3 >
  , stan::gm::binary\_op\_expr::result< T1, T2, T3, T4, T5 >
  , stan::gm::division\_expr::result< T1, T2, T3 >
  , stan::gm::elt\_division\_expr::result< T1, T2, T3 >
  , stan::gm::elt\_multiplication\_expr::result< T1, T2, T3 >
  , stan::gm::exponentiation\_expr::result< T1, T2, T3, T4, T5 >
  , stan::gm::expr\_type
  , stan::gm::expression\_as\_statement::result< T1, T2, T3 >
  , stan::gm::left\_division\_expr::result< T1, T2, T3 >
  , stan::gm::logical\_negate\_expr::result< T1, T2, T3 >
  , stan::gm::modulus\_expr::result< T1, T2, T3, T4 >
  , stan::gm::multiplication\_expr::result< T1, T2, T3 >
  , stan::gm::negate\_expr::result< T1, T2, T3, T4 >
  , stan::gm::remove\_loop\_identifier::result< T1, T2 >
  , stan::gm::scope\_lp::result< T1 >
  , stan::gm::set\_allows\_sampling\_origin::result< T1, T2, T3 >
  , stan::gm::set\_fun\_type2::result< T1, T2 >
  , stan::gm::set\_fun\_type3::result< T1, T2 >
  , stan::gm::set\_fun\_type::result< T1, T2 >
  , stan::gm::set\_fun\_type\_named::result< T1, T2, T3, T4, T5 >
  , stan::gm::set\_var\_type::result< T1, T2, T3, T4 >
  , stan::gm::set\_void\_function::result< T1, T2, T3, T4 >
  , stan::gm::subtraction\_expr3::result< T1, T2, T3 >
  , stan::gm::transpose\_expr::result< T1, T2 >
  , stan::gm::unscope\_locals::result< T1, T2 >
  , stan::gm::unscope\_variables::result< T1, T2 >
  , stan::gm::validate\_allow\_sample::result< T1, T2, T3 >
  , stan::gm::validate\_assignment::result< T1, T2, T3, T4 >
  , stan::gm::validate\_declarations::result< T1, T2, T3, T4 >
  , stan::gm::validate\_expr\_type3::result< T1, T2 >
  , stan::gm::validate\_int\_expr2::result< T1, T2, T3 >
  , stan::gm::validate\_int\_expr3::result< T1, T2 >
  , stan::gm::validate\_integrate\_ode::result< T1, T2, T3, T4 >
  , stan::gm::validate\_non\_void\_arg\_function::result< T1, T2, T3 >
  , stan::gm::validate\_non\_void\_expression::result< T1, T2, T3 >
  , stan::gm::validate\_return\_allowed::result< T1, T2, T3 >
  , stan::gm::validate\_return\_type::result< T1, T2, T3 >
  , stan::gm::validate\_sample::result< T1, T2, T3 >
  , stan::gm::validate\_void\_return\_allowed::result< T1, T2, T3 >
  , stan::is\_vector< T >
  , stan::is\_vector< const T >
  , stan::is\_vector< Eigen::Block< T > >
  , stan::is\_vector< Eigen::Matrix< T, 1, Eigen::Dynamic > >
  , stan::is\_vector< Eigen::Matrix< T, Eigen::Dynamic, 1 > >
  , stan::is\_vector< std::vector< T > >
  , stan::math::common\_type< T1, T2 >
  , stan::math::common\_type< Eigen::Matrix< T1, R, C >, Eigen::Matrix< T2, R, C > >
  , stan::math::common\_type< std::vector< T1 >, std::vector< T2 > >
  , stan::math::index\_type< const T >
  , stan::math::index\_type< Eigen::Matrix< T, R, C > >
  , stan::math::index\_type< std::vector< T > >
  , stan::math::pass\_type< T >
  , stan::math::pass\_type< double >
  , stan::math::pass\_type< int >
  , stan::math::promote\_scalar\_type< T, S >
  , stan::math::promote\_scalar\_type< T, Eigen::Matrix< S, 1, Eigen::Dynamic > >
  , stan::math::promote\_scalar\_type< T, Eigen::Matrix< S, Eigen::Dynamic, 1 > >
  , stan::math::promote\_scalar\_type< T, Eigen::Matrix< S, Eigen::Dynamic, Eigen::Dynamic > >
  , stan::math::promote\_scalar\_type< T, std::vector< S > >
  , stan::math::store\_type< T >
  , stan::math::store\_type< double >
  , stan::math::store\_type< int >
  , stan::math::value\_type< const T >
  , stan::math::value\_type< Eigen::Matrix< T, R, C > >
  , stan::math::value\_type< std::vector< T > >
  , stan::return\_type< T1, T2, T3, T4, T5, T6 >
  , stan::scalar\_type< T >
  , stan::scalar\_type< Eigen::Matrix< T, Eigen::Dynamic, Eigen::Dynamic > >
  , stan::scalar\_type< T \* >
  , stan::scalar\_type\_pre< T >
  , validate\_decl\_constraints::result< T1, T2, T3, T4 >
  , validate\_double\_expr::result< T1, T2 >
  , validate\_identifier::result< T1, T2 >
  , validate\_int\_data\_expr::result< T1, T2, T3, T4, T5 >
  , validate\_int\_expr::result< T1, T2, T3 >
- type\_
  : stan::gm::array\_literal
  , stan::gm::binary\_op
  , stan::gm::double\_literal
  , stan::gm::fun
  , stan::gm::index\_op
  , stan::gm::int\_literal
  , stan::gm::unary\_op
  , stan::gm::variable
- type\_identifier\_r
  : stan::gm::bare\_type\_grammar< Iterator >


---

[ Stan Home Page ]

*© 2011–2014,
Stan Development Team.*
